# Related bifunctional restriction endonuclease-methyltransferase triplets: TspDTI, Tth111II/TthHB27I and TsoI with distinct specificities

**DOI:** 10.1186/1471-2199-13-13

**Published:** 2012-04-10

**Authors:** Agnieszka Zylicz-Stachula, Olga Zolnierkiewicz, Arvydas Lubys, Danute Ramanauskaite, Goda Mitkaite, Janusz M Bujnicki, Piotr M Skowron

**Affiliations:** 1 Division of Theoretical Physical Chemistry, Department of Chemistry, University of Gdansk, Sobieskiego 18, 80-952 Gdansk, Poland; 2 Division of Environmental Molecular Biotechnology, Department of Chemistry, University of Gdansk, Sobieskiego 18, 80-952 Gdansk, Poland; 3 Thermo Fisher Scientific, V.A.Graiciuno 8, LT-02241 Vilnius, Lithuania; 4 Laboratory of Bioinformatics and Protein Engineering, International Institute of Molecular and Cell Biology in Warsaw, Ks. Trojdena 4, 02-109 Warsaw, and Institute of Molecular Biology and Biotechnology, Faculty of Biology, Adam Mickiewicz University, Umultowska 69, Poznan, Poland

## Abstract

**Background:**

We previously defined a family of restriction endonucleases (REases) from *Thermus *sp., which share common biochemical and biophysical features, such as the fusion of both the nuclease and methyltransferase (MTase) activities in a single polypeptide, cleavage at a distance from the recognition site, large molecular size, modulation of activity by S-adenosylmethionine (SAM), and incomplete cleavage of the substrate DNA. Members include related thermophilic REases with five distinct specificities: TspGWI, TaqII, Tth111II/TthHB27I, TspDTI and TsoI.

**Results:**

TspDTI, TsoI and isoschizomers Tth111II/TthHB27I recognize different, but related sequences: 5'-ATGAA-3', 5'-TARCCA-3' and 5'-CAARCA-3' respectively. Their amino acid sequences are similar, which is unusual among REases of different specificity. To gain insight into this group of REases, TspDTI, the prototype member of the *Thermus *sp. enzyme family, was cloned and characterized using a recently developed method for partially cleaving REases.

**Conclusions:**

TspDTI, TsoI and isoschizomers Tth111II/TthHB27I are closely related bifunctional enzymes. They comprise a tandem arrangement of Type I-like domains, like other Type IIC enzymes (those with a fusion of a REase and MTase domains), e.g. TspGWI, TaqII and MmeI, but their sequences are only remotely similar to these previously characterized enzymes. The characterization of TspDTI, a prototype member of this group, extends our understanding of sequence-function relationships among multifunctional restriction-modification enzymes.

## Background

Subtype IIS enzymes are a growing group of atypical REases that recognize a specific DNA sequence and cleave outside it at a defined distance, up to 21 nt, within any sequence [1,2]. Since their discovery, they have attracted considerable attention as objects of basic research in the field of protein-DNA interactions and as advanced tools for genetic engineering. One of the most intensively studied REases is FokI, specific to 5'-GGATG(N9/13)-3' sites, where asymmetry of the recognition site apparently imposes an unusual type of interaction with DNA: the large protein, monomeric in solution, transiently forms dimers and binds two recognition sites while the DNA loop is being generated [3]. Another subtype IIS REase - MmeI - not only cleaves DNA at 20/18 nt - one of the sites furthest removed from the recognition site - but also represents a model of a minimal restriction-modification system, where only one (the top) strand of the recognition site is methylated [4]. Molecular applications of subtype IIS enzymes, especially FokI, have been developed since the 1980s, including universal REase, cleaving DNA at a pre-programmed site [5-8], Achilles' Heel Cleavage [9,10], gene amplification [11], gene fusion [12], unidirectional DNA trimming [13], locating methylated bases in DNA [14], gene mutagenesis using excision linkers [1], and others [1,5-8]. Chandrasegaran et al. have developed a series of genetically engineered fusions of a non-specific C-terminal nuclease domain of FokI and specific DNA binding proteins, such as zinc-finger [15,16], *Ubx *homeodomain [17] or structure-specific Z-DNA nuclease [18]. Such artificial constructs have been used to rearrange mammalian genomes [16]. A recently discovered family of enzymes from *Thermus *sp. [19,20] belongs not only to subtype IIS, but also to subtypes IIC and IIG. These enzymes are bifunctional, with REase and MTase activities within a single polypeptide (Type IIC) and their cleavage is affected by SAM (subtype IIG). The experimentally characterized members of this family include TspGWI [5'-recognition sequence-3': ACGGA (11/9) [19], TaqII [GACCGA (11/9) [21]], TspDTI [(ATGAA (11/9) [20]], and the TsoI [TARCCA (11/9) [2]] as well as Tth111II/TthHB27I isoschizomer pair [(CAARCA (11/9) [2,22]]. The family shares several functional aspects, including a large molecular size of approximately 120 kDa (larger than typical REases and average-sized prokaryotic proteins, but similar to other subtype IIC enzymes [2]), similarity of amino acid sequences despite distinct specificities (unusual for REases), an identical cleavage distance of 11/9 nt, an acidic isoelectric point around 6 (except for TsoI), a domain structure related to simplified Type I REases, REase activity affected by SAM, and an origin from within the same genus *Thermus*, suggesting that they have evolved from one or a few common ancestors [19,20,23]. We recently reported for TspGWI enzyme a new type of substrate specificity change, induced by the replacement of SAM with its analogue - sinefungin (SIN) [24]. The chemically- induced recognition site relaxation changes the cleavage frequency of the REase from 5-bp to 3-bp. Such a molecular tool may be useful for generating quasi-random genomic libraries, as it is the second (after CviJI/CviJI*) most frequently cleaving REase [25]. In this paper we describe the cloning, expression and characterization of TspDTI, followed by a bioinformatics analysis of a subfamily of closely related enzymes (TspDTI, Tth111II/TthHB27I and TsoI), which appears to be distinct from the more remotely related sub-family that includes TspGWI and TaqII REases [23].

## Results and discussion

### Sequencing, cloning and expression of the *tspDTIRM *gene

In the course of studying the new *Thermus *sp. family of enzymes, we cloned the genes coding for TaqII, TthHB27I, TsoI (manuscripts in preparation) and TspGWI [23]. Initial data referring to TspDTI sequence we have previously deposited in GenBank (EF095489.1). In this work the sequencing data were confirmed and the TspDTI coding gene was *de novo *cloned into different expression system to improve protein yield. In our attempts to clone the *tspDTIRM *gene we experienced serious difficulties. Neither the biochemical selection for the methylation phenotype approach nor the 'white-blue' screen for DNA damage/modification resulted in the isolation of recombinant clones, which was also the case with *tspGWIRM *gene cloning [23]. Apparently, low enzymatic turnover of the enzymes of the *Thermus *sp. family, greatly reduced activity at 37°C and the incomplete cleavage of the plasmid DNA precluded positive results with the classic methods listed above, even though complete cleavage is not required for DNA damage detection in the 'white-blue' method. We therefore used a modification of the previously established, successful *tspGWIRM *cloning protocol ([23]; see Additional file 1). The protocol includes two stages: (*i*) a gene nucleotide sequence prediction starting from N-terminal and internal amino acid sequences of REase proteolytic fragments followed by PCR using degenerated and non-degenerated primers, and (*ii*) direct in-frame insertion of an amplified *tspDTIRM *gene into a strictly temperature-regulated *Escherichia coli (E.coli) *pACYC184-derived expression vector, containing a P_R_ bacteriophage lambda promoter and overexpressing the bacteriophage lambda thermolabile CI repressor. The system makes use of low temperature cultivation under permissive conditions (at ca 28°C), both of which prevent REase expression and suppress the activity of any leaking thermophilic REase.

**Figure 1:**
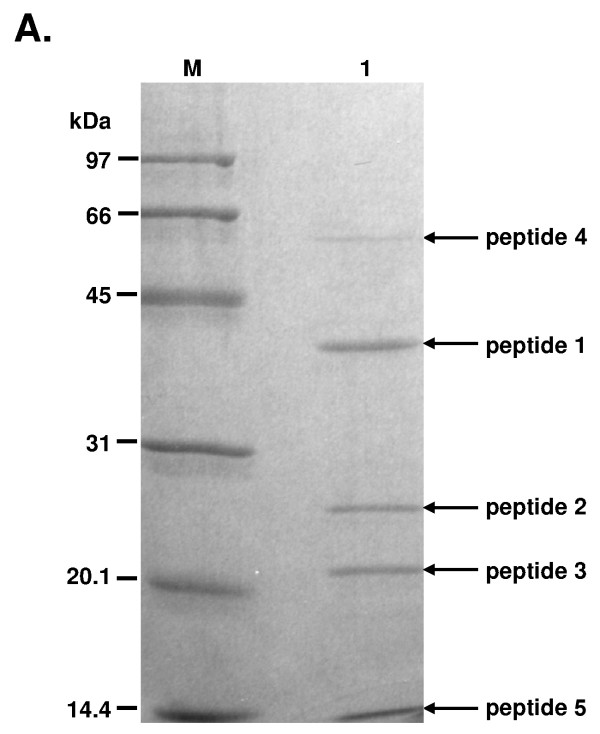
**Limited proteolytic digestion of TspDTI**. Purified native TspDTI was subjected to proteolytic digestion on immobilized TPCK-trypsin. Lane M, protein marker (GE Healthcare), bands marked: 97 kDa, phosphorylase b; 66 kDa, bovine serum albumin; 45 kDa, ovoalbumin; 31 kDa, carbonic anhydrase; 20.1 kDa, trypsin inhibitor; 14.4 kDa, lysozyme. Lane 1, proteolysis products. All the peptides obtained are marked with horizontal arrows. Of the 5 polypeptides obtained, only peptides 1, 2 and 3 were subjected to N-terminal sequencing.

The *tspDTIRM *gene nucleotide sequence was determined using an approach similar to that of TspGWI [23]; however, there are substantial differences in the execution of the method. The native TspGWI N-terminus could not be sequenced, so we had to initiate sequencing starting from internal proteolytic peptides and perform the PCR/sequencing divergently with degenerated primers, followed by non-degenerated ones. In contrast, native TspDTI N-terminus sequencing was not problematic. The sequencing of intact protein resulted in a long 35- amino acid stretch with a relatively good signal - MSPSREEVVAHYADRLHQVLQKTIAQNPNEAEFRR. In addition short internal 18-, and 12- amino acid sequences - LGAPVFSALAAADGGTLQ (peptide 1) and REPREPEFYGIMDIG (peptide 3) - were obtained from proteolytic fragments (Figure 1; Additional file 1). Based on the amino acid sequences, the primers were designed in most part arbitrarily, being founded on a back-translated amino acid sequence using codons, which were assumed to exist with the highest probability, as concluded from codon usage data from ORFs of *Thermus *sp. genes available in GenBank. The high GC content of *Thermus *genes (app. 70% GC) was also considered in codon selection, whenever applicable. Sets of combined primers were used to complete entire *tspDTIRM *ORF as well as short stretches of flanking regions (see the Methods section).

**Figure 2:**
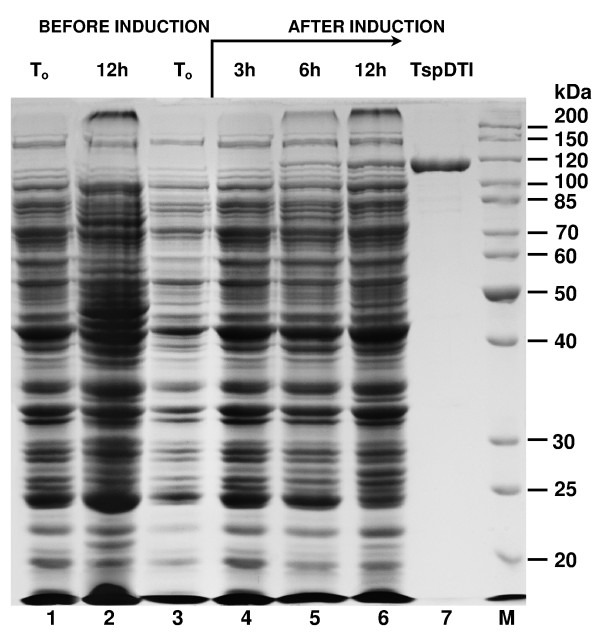
**SDS/PAGE analysis of the induction pattern of recombinant TspDTI endonuclease**. Lane 1, control culture - crude lysate from *E. coli *expressing the cloned *tspDTIRM *gene, without induction (OD_600 _= 0.7); lane 2, control culture after 12 h of cultivation; lane 3, crude lysate from *E. coli *expressing the cloned gene, before induction (OD_600 _= 0.7); lane 4, crude lysate from *E. coli *expressing the cloned gene 3 h after induction; lane 5, crude lysate 6 h after induction; lane 6, crude lysate 12 h after induction; lane 7, purified, homogeneous recombinant TspDTI protein; lane M, protein marker (Thermo Fisher Scientific/Fermentas).

The verified *tspDTIRM *ORF was cloned into a P_R_ promoter vector and subjected to *E. coli * expression optimization experiments (data not shown). Recombinant TspDTI protein was purified using a 6-step procedure, with the protein expression optimized in *E. coli *(Figure 2). Interestingly, in spite of the cloning being under the control of a strong P_R_ promoter, the protein becomes detectable in the induced cells after only 3 h of growth under non-permissive conditions, and keeps accumulating until the late stationary phase, even after 12 h cultivation at 42°C. This is probably due to the combination of the following factors: (*i*) the GC rich ORF sequence distant from *E. coli *optimum codon usage, (*ii*) the slow transcription of the GC-rich *tspDTIRM *gene, (*iii*) the presence of numerous hairpin structures within the gene, and (*iv*) the very large size of the protein to be translated. Nevertheless, optimization of expression culture growth/induction conditions yielded adequate amounts of TspDTI (about 0.4 mg of the protein per litre of bacterial culture).

### Properties of the *tspDTIRM *gene

**Figure 3:**
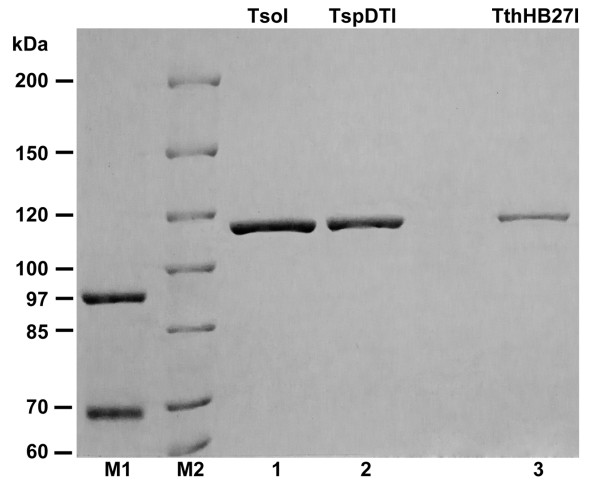
**SDS/PAGE comparison of purified, recombinant homogeneous TspDTI, TsoI and TthHB27I (extended run on 6% gel)**. Lane M1, protein marker (GE Healthcare); lane M2, protein marker (Thermo Scientific); lane 1, TsoI; lane 2, TspDTI, lane 3, TthHB24I.

The *tspDTIRM *gene ORF coding for the REase-MTase bifunctional protein is 3339 bp in length coding for the 1112 amino acid polypeptide [GenBank: EF095489, ABO26711]. The calculated molecular weight of the TspDTI is 126 885 Da, atypically large for a prokaryotic protein. The sizes of the *Thermus *sp. family enzymes were compared and shown to match the estimation from the SDS/PAGE (Figures 2 and 3) and molecular sieving of the native protein [20], indicating its monomeric organization, just like other *Thermus *sp. family members (Table 1). The calculated isoelectric point is 6.68, indicating that TspDTI is a slightly acidic protein. Typically, REases and other DNA-interacting proteins are rather basic proteins. The low pI is associated with 5 out of 6 *Thermus *sp. family enzymes: TspDTI, Tth111II, TthHB27I, TaqII and TspGWI. Only TsoI is moderately basic, with a calculated pI of 8.11 (Table 1). No sequence similarity of TspDTI to any MTase or DNA-binding protein was found in the flanking regions of the TspDTI ORF. The ORF begins with the ATG START codon and contains 3 putative upstream RBSs: -8 AG, -11 AGAAA and -18 GGA (see Additional file 2). The ORF is GC rich (57.99%); however, it is markedly lower than the *tspGWIRM *gene (69.19%) (GenBank: EF095488, ABO26710) and TaqII (Table 1 GenBank: AY057443, AAL23675) (Table 1) and other *Thermus *genes [20,26], suggesting that *tspDTIRM *might have been acquired/evolved differently, at least diverging at a later stage, which may have included horizontal gene transfer from a non-related bacteria.

**Table 1:** Summary of features of the *Thermus *sp.enzyme family

REase	TspDTI	Tth111II	TthHB27I	TsoI	TspGWI	TaqII
**Bacterial host**	*Thermus *sp. DT	*Thermus thermophilus *111	*Thermus thermophilus *HB27	*Thermus scotoductus*	*Thermus *sp. GW	*Thermus aquaticus *YTI

**Recognition and cleavage site**	ATGAA(11/9)	CAARCA(11/9)	CAARCA(11/9)	TARCCA(11/9)	ACGGA(11/9)	GACCGA(11/9)

**Recognition site length (bp)**	5	6, (degenerated)	6 (degenerated)	6 (degenerated)	5	6

**ORF GC%**	57.99	61.67	61.59	56.54	69.19	66.27

**Polypeptide length (aa)**	1112	1106	1121	1116	1097	1106

**Molecular weight (kDa)**	126.885	125.956	127.672	126.474	120.202	125.675

**Native molecular size (kDa)/protein organization**	110-130 monomer	110-130 monomer	ND	ND	110-130 monomer	110-130 monomer

**Optimum reaction temperature**	65°C	65-70°C	ND	55°C	65°C	65°C

**Single site cleavage**	Yes	ND	ND	ND	Two sites preferred	Yes

**Isoelectric point (calculated)**	6.68	6.60	6.80	8.11	6.58	5.40

**Specific DNA **+ **methylation**		ND	ND	+	+	+

**Cleavage stimulation with SAM**	Yes	ND	ND	Yes	Slight inhibition	Yes

**Cleavage stimulation with SIN (SAM analogue)**	Yes	ND	ND	ND	Yes, specificity change	Yes, specificity change

**Methylation stimulation with **Ca^2+^	No	ND	ND	ND	Yes	Yes

**Cleavage stimulation with divalent cations**	Ca^2+^	ND	ND	ND	Mn^2+^, Fe^2+^, Co^2+ ^not stimulated by Ca^2+^	ND

Domain organization*	R-Mh-Mc-S	R-Mh-Mc-S	R-Mh-Mc-S	R-Mh-Mc-S	R-Mh-Mc-S	R-Mh-Mc-S

Cleavage catalytic motif (B/E)**	atypical D-EXE (B)	atypical D-EXE (B)	atypical D-EXE (B)	atypical D-EXE (B)	PDX_13_EX_1_K (B + E)	PDX_13_EX_1_K (B + E)

SAM binding motif: (B/E)**	DPACGSG (B)	DPACGSG (B)	DPACGSG (B)	PPACGSG (B)	DPAVGTG (B + E)	DPAMGTG (B + E)

Methylation catalytic motif (B/E)**	NPPW (B)	NPPW (B)	NPPW (B)	NPPW (B)	NPPY (B + E)	NPPY (B + E)

Subfamily homology*	Subfamily TspDTI	Subfamily TspDTI	Subfamily TspDTI	Subfamily TspDTI	Subfamily TspGWI	Subfamily TspGWI

**Reference**	[20]	GenBank: AY726624.1, [20,26]	GenBank: AE017221.1	This work, manuscript in preparation	[19,23]	GenBank: AY057443, 21

* Domain organization: R, REase catalytic domain; Mh, MTase helical domain; Mc, MTase catalytic domain; S, putative specific DNA recognition domain

** B, determined by bioinformatics analysis; E, experimentally determined by site-directed mutagenesis.

### Bioinformatics analyses of TspDTI: Prediction of domains and functional motifs

**Figure 4:**
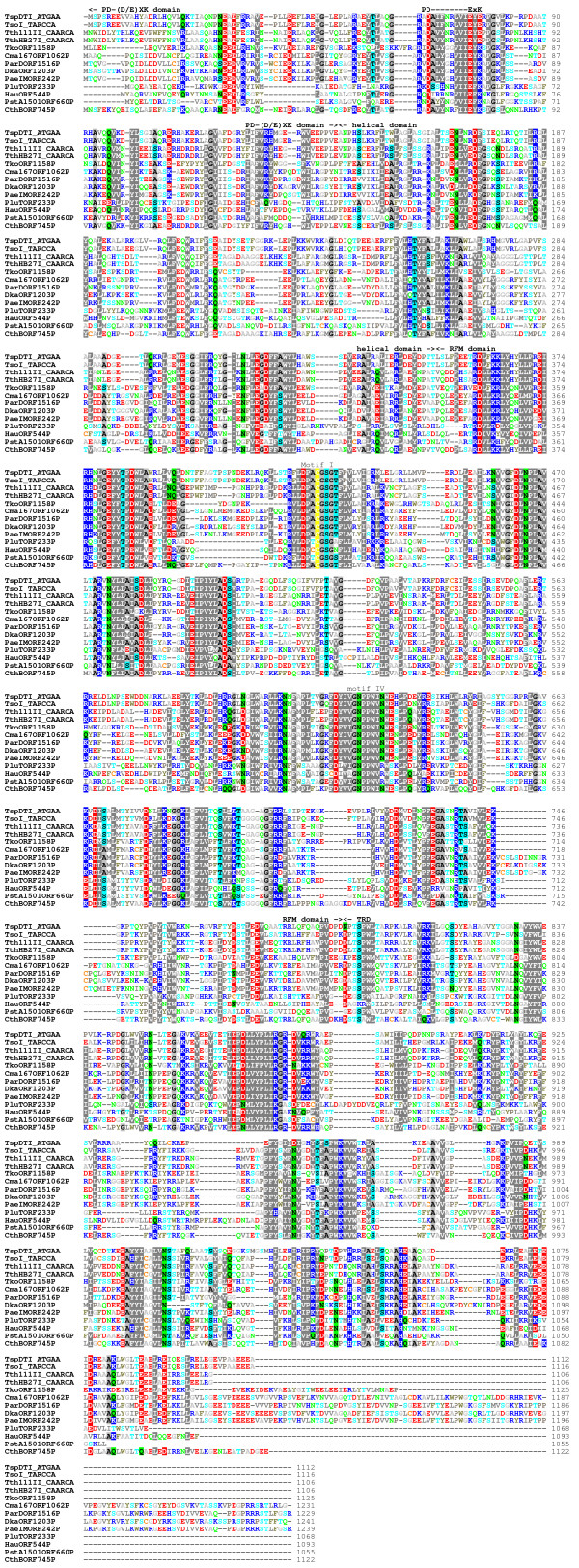
**Sequence alignment between TspDTI and its close homologues in REBASE (BLAST E-value < 1E-40)**.

Isolation and sequencing of the *tspDTIRM *gene revealed the predicted amino acid sequence of the encoded protein. Searches of REase sequences deposited in REBASE exhibited an overall similarity to a number of genuine and putative Type IIC enzymes, including the previously characterized nucleases TthHB27I and Tth111II (BLAST e-value 0, alignment covering essentially the whole protein length). Despite very high sequence similarity, these two enzymes exhibit different sequence specificity (CAARCA) [2,22] than TspDTI. Interestingly, two other Type IIC enzymes from *Thermus*, i.e. TspGWI (GenBank: EF095488, ABO26710) and TaqII (GenBank: AY057443, AAL23675), showed very low sequence similarity to TspDTI in pairwise comparisons (BLAST e-value 0.001 limited to a very short region of ~75 residues) and were thus excluded from the alignment (Figure 4).

Further bioinformatics analyses, in particular the comparison of sequence profiles, which is more sensitive to pairwise sequence comparisons (see Methods), showed that the central and C-terminal regions of TspDTI (aa ~370-1050) exhibit significant similarity to DNA:m^6^A MTase M.TaqI, whose structure is known (HHSEARCH e-value 0). M.TaqI belongs to the γ-class of DNA:m^6^A MTases, which is characterized by the following primary structure: the N-terminal catalytic Rossmann-fold MTase (RFM) domain with the order of motifs: X-I-II-III-IV-V-VI-VII-VIII, followed by the DNA binding domain, the so-called 'target recognition domain' (TRD) in the C-terminus [27,28]. The alignment between TspDTI and M.TaqI spanned both RFM and TRD domains. The N-terminal region of the TspDTI sequence, which extends beyond the region of homology to M.TaqI, exhibited a limited sequence similarity (HHSEARCH e-value 0.087) only to the HSDR_N family, which belongs to the PD-(D/E)XK superfamily of nucleases (accession number pfam04313/g in the PFAM database). Further, a multiple sequence alignment of TspDTI homologues revealed the presence of a candidate PD-(D/E)XK motif (Figure 4), resembling the active site of many REases and other nucleases [29]. Thus, TspDTI appears to comprise domains homologous to known nuclease and DNA:m^6^A MTase catalytic domains, and to the 'TRD' domain characteristic of γ-class DNA:m^6^A MTases.

To confirm the sequence-based predictions, we carried out a protein fold-recognition (FR) analysis (see Methods) with the aim of predicting the structures of individual domains in TspDTI. Since the FR method is designed to identify remote homology and predict structure for domain-size sequence fragments (20-500 aa), the TspDTI sequence was split into a series of overlapping segments and submitted to the GeneSilico metaserver [30]. FR analysis of the TspDTI sequence confirmed the existence of enzymatic and DNA-recognizing domains predicted by sequence analysis, albeit with low scores for the N-terminal and C-terminal domains (Table 2). Structure prediction also revealed the presence of a helical linker between the PD-(D/E)XK and RFM domains. The sequence of this region alone displayed no significant similarity to any known protein domain. When this region was analysed together with the neighbouring RFM domain, some of the fold-recognition methods proposed Type I enzyme structures as templates, with the alignment spanning both the RFM domain (present in all DNA MTases), and the helical domain characteristic only of Type I enzymes, which is involved in mediating protein-protein interactions [31]. However, the alignment of the helical linker regions of TspDTI and Type I MTases was too poor to establish with confidence whether they have similar tertiary structures.

**Table 2:** Bioinformatics analysis of TspDTI domains and functional motifs

Residues	domain	HHPRED	PCONS score	predicted function
1 ~ 160	PD-(D/E)XK	1m0d (Endo I) 21	1avq (λ exonuclease) 0.18	Mg^2+^-binding/DNA cleavage

~160-360	helical linker	no match	no match	interactions between domains

~360-790	RFM	2ar0 (M.EcoKI) 4.6e-36	2adm (M.TaqI) 1.9	SAM-binding/methylation

~790-	TRD	1ih2 (M.TaqI) 1.9e-13	1yf2 (S.MjaORF132P) 0.374	DNA-binding

**Figure 5:**
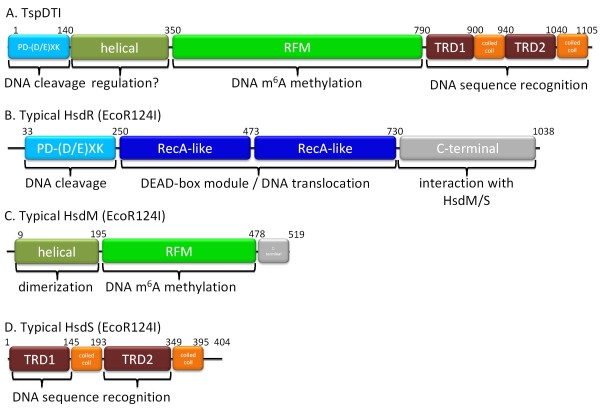
**Schematic organization of the domains of the bifunctional TspDTI enzyme**. (**A**) TspDTI domain architecture. (**B, C**, and **D**) Typical HsdR, HsdM, and HsdS subunit domain architecture, illustrated by way of example with EcoR124I.

Combined sequence analysis and structure prediction (Figures 4 and 5) enable us to propose the key functional residues of TspDTI. In the PD-(D/E)XK domain, the putative catalytic residues are D66, E75, and E77. Thus, the nuclease domain of TspDTI exhibits an atypical D-EXE pattern, which has been observed previously, e.g. in the R.BamHI enzyme [32]. Interestingly, the same pattern is present in the nuclease active sites of TthHB27I, Tth111II and TsoI (see Additional file 3), while other homologues of TspDTI (putative nucleases) exhibit the typical D-EXK pattern (Figures 4 and 5). In the RFM domain, the SAM-binding site includes the carboxylate residues D422, D464, and D500 (from motifs I, II and III respectively), while the target adenine-binding site includes the NPPW626 tetrapeptide (motif IV) and F732 (motif VIII). At this stage of the analysis, the details of sequence-specific DNA recognition by TspDTI cannot be predicted. However, based on the identification of homologous loops in the sequence of TspDTI and in the protein-DNA complex of M.TaqI, we suggest that the following regions may harbour specificity determinants of TspDTI: WTRLAK968, PQET987 and KSMGS1028. In accordance with this prediction, while the corresponding regions in TthHB27I and Tth111II (enzymes that recognize a DNA sequence other than TspDTI) possess significantly different amino acid residues, they show greater similarity to TspDTI in surrounding regions that are not expected to make direct contact with the DNA. The testing of these predictions, however, is beyond the scope of this article.

Summarizing, the results of the bioinformatics analyses suggest that TspDTI is a fusion protein comprising a tandem arrangement of the following domains: a PD-(D/E)XK nuclease domain, a helical domain, an RFM MTase domain and a TRD substrate-binding domain (Figure 5). This domain organization resembles the structure of the recently analysed Type IIC enzymes TspGWI [19] and MmeI [33]), to which TspDTI exhibits only very limited sequence similarity, restricted primarily to the central RFM domain (data not shown). The presence of related domains in a common linear arrangement suggests that all these enzymes evolved from a common ancestor, but that they diverged greatly in all regions except the RFM domain. This analysis indicates a low sequence similarity between TspDTI and TspGWI, dividing the *Thermus *sp. family group into two subfamilies: TspDTI, TsoI (see Additional file 3) and Tth111II/TthHB27I ('subfamily TspDTI') as opposed to TspGWI and TaqII REases ('subfamily TspGWI') [23]. In spite of the remote sequence similarities between REases, certain structural analogies are emerging. Particularly, based on the crystal structure of a Type IIG bifunctional enzyme BpuSI, an alpha-helical domain that connects the endonuclease and MTase domains has been suggested to regulate and physically couple their relative conformations and activities and that it may establish the cleavage distance from the enzyme's target site [34]. The helical domain in TspDTI and its relatives is likely to fulfil a similar role as its counterpart in BpuSI.

### Enzymatic properties of TspDTI

Native and recombinant TspDTI proteins were purified to homogeneity ([20]; Figure 2) and used to study the biochemical features and reaction conditions of DNA cleavage and methylation activity of the enzyme. The apparent molecular mass of the native protein under denaturing conditions was found to be 114.5 kDa [20], corresponding to the molecular mass of cloned TspDTI isolated from *E. coli *DH11S [pRZ-TspDTI] ([20]; Figure 2). A comparative assay of recognition specificity, cleavage distance and reaction buffer requirements of both enzymes revealed no difference (not shown). Controlled purification from *E. coli *(limited to the first chromatographic step), devoid of pRZ-TspDTI, did not show any DNA cleaving activity (not shown). The molecular mass was evidently very similar between three members (TspDTI, TsoI and TthHB27I) of the 'TspDTI subfamily', as only prolonged SDS/PAGE showed a slight differentiation between the enzymes (Figure 3). Recombinant TspDTI was also subjected to analytical gel filtration in a buffer with a composition close to the physiological, containing 3 mM MgCl_2_ (in the absence of DNA), using conditions described previously [20]. The experiment showed that the recombinant REase behaved like a monomer, just as the native TspDTI (Table 1).

**Figure 6:**
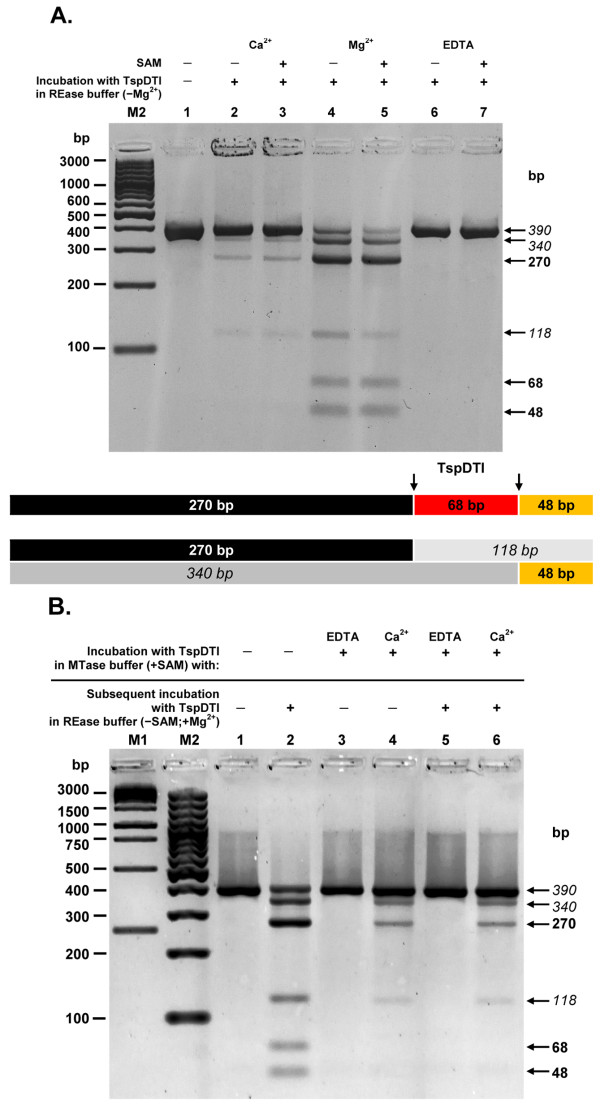
**Bifunctionality of TspDTI: restriction and methylation activities of the enzyme**. (**A**) Effect of divalent metal cations on restriction activity of TspDTI. The DNA substrate used contains two TspDTI sites (→→). Partial digestion bands are marked in italics. Samples of 7.8 pmol 390 bp PCR fragment were incubated with an excess of TspDTI REase for 1 h at 70°C in 'primary TspDTI REase' buffer devoid of Mg^2+ ^(10 mM Tris-HCl pH 8.0, 1 mM DTT) in the presence or absence of 50 μM SAM. The reaction buffer was supplemented with EDTA, Mg^2+ ^or Ca^2+ ^ions. Lane M2, 100 bp DNA ladder (selected bands marked); lane 1, undigested 390-bp PCR fragment; lane 2, incubation with TspDTI and Ca^2+^; lane 3, incubation with TspDTI, Ca^2+ ^and SAM; lane 4, incubation with TspDTI and Mg^2+^; lane 5, incubation with TspDTI, Mg^2+ ^and SAM; lane 6, incubation with TspDTI and EDTA; lane 7, incubation with TspDTI, EDTA and SAM. (**B**) MTase activity of TspDTI. The DNA substrate used contains two TspDTI sites (→→). Partial digestion bands are marked in italics. Samples of 7.8 pmol 390 bp PCR fragment were incubated with 78 pmol TspDTI protein in the TspDTI MTase buffer (10 mM Tris-HCl, pH 8.0, 1 mM DTT, 200 μM SAM) in the presence of either EDTA or Ca^2+ ^ions. Proteins were removed by proteinase K digestion. The resulting DNA was purified and challenged with an excess of TspDTI REase for 1 h at 70°C in the 'primary TspDTI REase' buffer (10 mM Tris-HCl pH 8.0, 1 mM DTT) supplemented with 10 mM MgCl2; Lane M1, 1 kb DNA ladder (selected bands marked); lane M2, 100 bp DNA ladder (selected bands marked); lane 1, undigested 390-bp PCR fragment; lane 2, incubation of PCR fragment with TspDTI in REase buffer; lane 3, incubation with TspDTI in MTase buffer + EDTA/no subsequent incubation; lane 4, incubation with TspDTI in MTase buffer + Ca^2+^/no subsequent incubation; lane 5, incubation with TspDTI in MTase buffer + EDTA/subsequent incubation with TspDTI in REase buffer; lane 6, incubation with TspDTI in MTase buffer + Ca^2+^/subsequent incubation with TspDTI in REase buffer.

We showed previously that the temperature activity range extended from 42°C to 85°C (10% or more activity), with the maximum observed at 65-75°C, while a 20 min incubation at 89°C deactivated the enzyme. Incubation at 37°C resulted in approx. 5% activity. The optimal ionic strength is in Tris-HCl buffered (pH 8.0-8.5) MgCl_2_ solution, without any added salt [20]. As expected, TspDTI maintains the absolute requirement for Mg^2+ ^for cleavage activity. Remarkably, the effect of Ca^2+ ^ions differs from that of TspGWI [23]. TspGWI MTase activity is strongly stimulated by Ca^2+ ^and SAM, whereas restriction activity is not supported [23]. Compared with the effect of Mg^2+ ^ions, TspDTI restriction activity is stimulated by Ca^2+ ^ions, but to a lesser extent and there is no difference in the digestion patterns in the presence and absence of SAM (Figure 6A, lanes 2 and 3).

Essentially the same TspDTI digestion patterns were observed, regardless of whether the substrate DNA was incubated with enzyme and Ca^2+ ^ions in the MTase buffer only or subjected to subsequent cleavage with TspDTI in the presence of Mg^2+^, following previous incubation with the enzyme and Ca^2+ ^ions (Figure 6B, lanes 4 and 6). These results indicate that after the incubation of TspDTI and substrate DNA in the MTase buffer with Ca^2+ ^ions, the TspDTI cannot further cleave such DNA, even though the Ca^2+^/TspDTI-treated DNA is carefully purified and subjected to subsequent incubation with an excess of TspDTI in the optimal TspDTI REase buffer supplemented with Mg^2+ ^ions (Figure 6B, lanes 4 and 6). The observed predominance of 'resistant' DNA indicates that Ca^2+ ^ions: (*i*) do not inhibit MTase activity, while stimulating REase only marginally, or else (*ii*) stimulate both enzyme activities, with a bias towards methylation activity. Hence, it is possible that after the incubation both the restriction and methylation processes are completed, leaving the substrate DNA either cleaved or methylated. The observed effect could also be explained by the existence of methylation-independent (unmodified), REase resistant sites. Such possibility, however, is rather remote, as in comparison to the Ca^2+^/TspDTI-treated DNA (Figure 6B, lane 6) the previously non-incubated substrate DNA, subjected to cleavage in the presence of Mg^2+ ^ions, is cut to a greater extent (Figure 6A, lanes 4-5; Figure 6B, lane 2).

**Figure 7:**
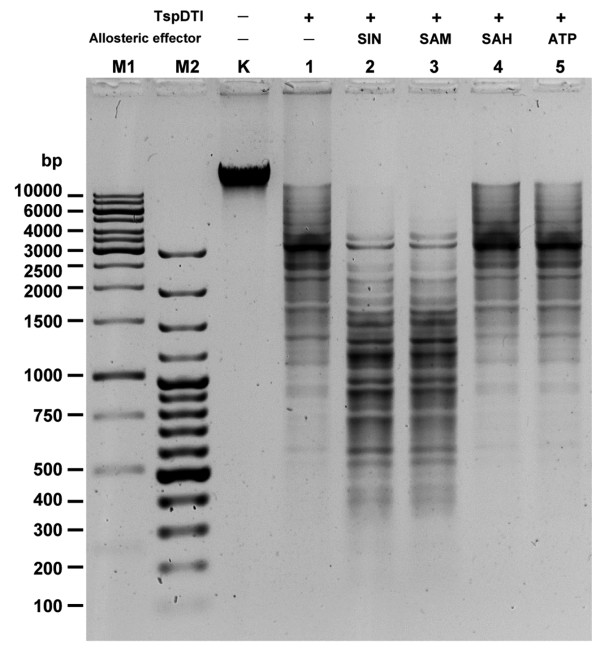
**Effect of allosteric cofactors on TspDTI REase activity**. 1 μg (= 5.58-pmol restriction sites) bacteriophage λ DNA was digested with 2.79 pmol TspDTI (0.5:1 M ratio of enzyme to recognition sites) for 30 min at 70°C and electrophoresed on 1.2% agarose/TBE gel. Lane M1, 1 kb ladder (selected bands marked); lane M2, 100 bp ladder (selected bands marked); lane K, undigested λ DNA; lane 1, (+ TspDTI, no allosteric cofactor; lane 2, (+ TspDTI, + 50 μM SIN); lane 3, (+ TspDTI, + 50 μM SAM); lane 4, (+ TspDT, + 50 μM SAH); lane 5, (+ TspDTI, + 50 μM ATP).

These results corroborate those of enzymology investigations into the mode of action of another subclass IIG/IIC bifunctional enzyme Eco57I (Lubys Arvydas, personal communication). This may reflect differences in the structure of the catalytic sites. In the standard restriction buffer with Mg^2+ ^ions TspDTI restriction activity is not stimulated by S-adenosylhomocysteine (SAH) and ATP (Figure 7, lanes 4 and 5), but is stimulated equally by both SAM and its analogue - SIN, which is not a methyl group donor (Figure 7, lanes 2 and 3). This leads to the conclusion that both restriction and methylation activities are SAM-stimulated. Nevertheless, it is important to note that the enzymes of the *Thermus *sp. family exhibit a spectrum of responses to SAM, which makes this group interesting. TspGWI restriction activity is actually slightly inhibited by SAM [23]. The observed negative response suggests that the enzyme is still capable of binding SAM. But then, conformational allosteric stimulation of TspGWI is somehow anti-functional. Alternatively, the effect described above may be associated with two competing reactions: DNA restriction and methylation. The addition of SAM may shift the equilibrium of the reactions in favour of DNA methylation. On the other hand, the inhibition of REase activity by SAM may play a role in the regulation of TspGWI REase versus MTase activities *in vivo*. Two analogues of SAM - SIN with charge distribution reversed compared to SAM and SAH - exert a very different influence on TspDTI (Figure 7, lanes 2 and 4). SIN stimulates REase activity, which also suggests that the *Thermus *sp. family enzymes may have two physically separate binding sites for SAM: one for allosteric stimulation of the REase activity and another one for typical SAM binding/methylation. Alternatively, binding to a single SAM-specific protein region may induce a conformational change in this large protein, also affecting the distant REase catalytic domain, so that cleavage activity is enhanced several times. Another possibility is that SIN, being an analogue mimicking an 'unreacted' methyl group donor (SAM), causes the enzyme to maintain a conformation different from that maintained in the presence of the MTase reaction product - SAH. These results also suggest a mixed type of mutual dependence of REase and MTase activities: while autonomous enough to perform functions independently, some sort of intertwined communication still occurs between functional domains. We managed to separate REase and MTase activities using site-directed mutagenesis of the genes encoding TspGWI and TaqII enzymes ([23], manuscript in preparation). Since the recombinant *tspDTIRM *gene alone was cloned into *E. coli *without an additional MTase (which has thus far not been found) and stably maintained at 28°C, it is possible that built-in MTase activity is sufficient to protect the recombinant host DNA from autorestriction. Moreover, a much reduced restriction activity < 5% at low temperature and the presence of cellular SAM appear to favour methylation *in vivo*.

Further research is needed to evaluate the existence of a separate MTase, contributing to the overall TspDTI modification activity and SAM influence. The gene encoding such an MTase may be located at a greater distance than the flanking regions of the *tspDTIRM *gene sequenced so far.

## Conclusions

(*i*) The modified protocol for cloning thermophilic REases was applied.

(*ii*) The *tspDTIRM *gene coding for 126.9 kDa TspDTI was sequenced and cloned.

(*iii*) Active bifunctional REase-MTase protein was expressed in *E. coli *and purified to homogeneity.

(*iv*) Bioinformatics studies predicted REase and MTase binding/catalytic motifs: the atypical D-EXE pattern as opposed to the TspGWI/TaqII PD-(D/E)XK pattern, DPACGSG and NPPW, and showed a modular structure of TspDTI.

(*v*) The TspDTI/TsoI/Tth111II/TthHB27I subfamily was differentiated using a bioinformatics comparison with the TspGWI-TaqII subfamily.

## Methods

### Bacterial strains, plasmids, media and reagents

*Thermus sp*. DT was obtained from Piotr Skowron's collection. The optimum cultivation conditions were a temperature of 60°C in a modified Luria broth (0.5% tryptose; 0.3% yeast extract; 0.2% NaCl; 0.001% dilution of 2.1 g/L stock Nitsch's trace elements; pH 7.2). The bacteria were harvested at OD600 = 1.6.

*E.coli *DH11S {*mcrA *Δ[*mrrhsdRMS*(rK-, mK+)-*mcrBC*] Δ(*lac-proAB*) Δ(*recA1398*) *deoR, rpsL, srl-thi, supE*/*F*' *proAB *+ *lacI*Q*Z*Δ*M15*} (Life Technologies, Gaithersburg, MD, USA) was used for all procedures. In most cases bacteria were grown in 2× yeast extract/tryptone (YT); for protein expression, bacteria were cultivated in Terrific Broth (TB) medium [35]. Media were supplemented with chloramphenicol (40 mg/mL) and 0.2% maltose. Difco media were from Becton- Dickinson (Franklin Lakes, NJ, USA), phosphocellulose P11 resin was from Whatman (Springfield Mill, UK) and hydroxyapatite HTP from Bio-Rad Laboratories (Hercules, CA, USA). Other chromatographic resins were from GE Healthcare (Uppsala, Sweden). Immobilized TPCK-trypsin and the BCA Protein Assay Reagent Kit were supplied by Pierce (Rockford, IL, USA). The miniprep DNA purification kit, *Sma*I endonuclease, T4 DNA polymerase, proofreading Taq DNA Polymerase blend (OptiTaq), Perfect Perfect 100-bp ladder and Perfect Plus 1-kb ladder were from Eurx Molecular Biology Products (Gdańsk, Poland). T4 polynucleotide kinase, antarctic phosphatase, BspHI and SalI REases were from New England Biolabs. Protein standards were from GE Healthcare (Uppsala, Sweden) and Thermo Fisher Scientific/Fermentas (Vilnius, Lithuania).

The cloning vectors pAPS (Cm^R^, MCS, col E1 *ori, f*1 *ori*, P_lac_ and T7 promoters) and pRZ4737 (Cm^R^, P15A *ori, f*1 *ori*, PR promoter) were from Bill Resnikoff [36] and further modified. λ DNA and plasmids pBR322 and pUC19 were from Vivantis (Shah Alam, Malaysia). T4 DNA ligase was from Epicenter (Madison, WI, USA). The DNA sequencing and PCR primer synthesis were performed at Genomed (Warsaw, Poland). All other reagents were purchased from Sigma-Aldrich (St. Louis, MO, USA).

### Native TspDTI purification and proteolysis of TspDTI and amino acid sequence determination

The native TspDTI enzyme was isolated from *Thermus *sp. DT as described previously [20]. Purified native TspDTI was subjected to limited TPCK-trypsin digestion to obtain internal polypeptides. Proteolysis of TspDTI was conducted in buffer T (20 mM Tris-HCl pH 8.3, 25 mM KCl, 3 mM MgCl_2_, 5% glycerol, 0.05% Tween 20, 0.5 mM DTT) with gel-immobilized TPCK-trypsin with shaking at 24°C for 3 h. The immobilized TPCK-trypsin was removed by centrifugation. Purified native TspDTI and the supernatant, containing TspDTI protein fragments were run on 10% SDS/PAGE denaturing gel and electroblotted onto a PVDF membrane in 100 mM CAPS-NaOH buffer pH 11.0. The N-terminal amino acid sequence analysis of polypeptides was performed on a gas-phase sequencer (Model 491, Perkin Elmer-Applied Biosystems). The phenylthiohydantoin derivatives were analysed by on-line gradient high performance liquid chromatography on a Microgradient Delivery System Model 140 C equipped with a Programmable Absorbance Detector Model 785A and Procise software (Perkin Elmer-Applied Biosystems).

### Determination of the nucleotide sequence and cloning of the *tspDTIRM* gene

The gene nucleotide sequence was obtained using a combination of PCR employing degenerated and non-degenerated primers (Additional file 1). In the first step, sets of degenerated/arbitrary primers, forming alternative pairs, were designed. The primers were designed arbitrarily on the basis of a back-translated amino acid sequence using codons, as concluded from codon usage data from ORFs of *Thermus *sp. genes and assumed from the high GC content of *Thermus *genes (app. 70% GC). A 105 bp *tspDTIRM *gene fragment was amplified with primers designed on the basis of 35 amino acid N-terminal sequences of TspDTI (Additional file 1). The forward primer 5'-ATGT(GC)CCCCTCCCGGGAGGAGGT(GC)GT(GC)GC(GC)CACTA-3' and the reverse primer 5'-CCG(GC)CGGAACTC(GC)GCCTCGTTGGGGTTCTG-3' were used. PCR was performed using an Applied Biosystems 2720 thermocycler in 100 μl of a reaction mixture containing 10 mM Tris-HCl pH 9.1, 50 mM KCl, 1.5 mM MgCl_2_, 0.1% Triton X-100, 6% formamide, 100 ng *Thermus *sp. DT genomic DNA, 0.25 μM of each primer, 100 μM of each dNTP and 5 U Taq DNA Polymerase. The cycling conditions employed a denaturation step of 3 min at 97°C, followed by the addition of the Taq DNA Polymerase at 85°C and 30 cycles of 30 s denaturation at 95°C, 30 s annealing at 55°C and 1 min elongation at 72°C. A 105 bp PCR product was agarose gel isolated and cloned into the pAPS vector at the SmaI site and sequenced. Insert sequencing established an internal 50 bp native sequence, non-primer modified, and thus used as a new non-degenerated primer (FdtN-ter: 5'-TGACAGGCTTCACCAAGTTCTTCAGAAAACCA-3') anchor for amplification with downstream degenerated/arbitrary reverse primers (Additional file 1).

The downstream portion of the *tspDTIRM *gene was obtained using amino acid sequences of internal proteolytic fragments. Homogeneous native (isolated from *Thermus *sp. DT) [20] TspDTI was subjected to limited proteolysis (Figure 1). Digestion yielded five bands of a stable partial digestion pattern (with limiting amounts of the protease used) (Figure 1). Three of the five bands of approximate sizes 60, 35, 25, 21 and 14.4 kDa were subjected to protein sequencing (Figure 1, peptide 1, 2 and 3) and yielded short internal 18-, and 12- amino acid sequences: LGAPVFSALAAADGETLQ (peptide 1) and REPEFYGIMDIG (peptide 3). Two reverse primers, designed on the basis of the amino acid sequences obtained - 1RDTpep1 5'-TCGGCGGCGGCGAGGGCGCTGAACAC-3' and 1RDTpep3 5'-CC(GT)AT(GA)TCCAT(GT)AT(ACGT)CCGTAGAACTC(GT)GGCTCCC-3' - resulted in PCR products of approx. 800 bp and 2800 bp (Additional file 1). These DNA fragments were cloned into the pAPS vector at the SmaI site and sequenced.

Combination of both standard PCR and the 'promiscuous PCR' setup yielded a sequence of 3943 bp contig with the complete *tspDTIRM *gene. Each strand was re-sequenced from *de novo *amplified entire contig using a *Thermus *sp. DT genomic DNA template. Regions containing discrepancies were sequenced several times under various conditions.

#### Analysis of nucleotide sequences

DNA sequences were obtained using the ABI Prism 310 automated sequencer with the ABI Prism BigDye Terminator Cycle Sequencing Ready Reaction Kit (Perkin Elmer Applied Biosystems, Foster City, CA, USA). The sequence data were analysed using ABI Chromas 1.45 software (Perkin Elmer Applied Biosystems) and DNASIS 2.5 software (Hitachi Software, San Bruno, CA, USA).

Overexpression of the TspDTI enzyme employed the modified vector pRZ4737 [36], a derivative of pACYC184 plasmid [37], carrying a lambda DNA section with the PR promoter under the control of the CI thermolabile repressor. The *cI *gene was located on the pRZ4737 backbone, allowing for host-independent expression in *E. coli*.

The *tspDTIRM *gene was PCR amplified with proofreading Taq-Pfu DNA polymerase using the oligonucleotides 5'-CGCCATGGCGAGCCCTTCCAGGGAAGAAGTTGTTG-3' and 5'-CAAAGATAATTTCGTCGACCCGCTCCTCTTC-3', which introduced the NcoI recognition site (underlined) at the 5'-end and the SalI recognition site (underlined) after the *tspDTIRM *gene STOP codon at the 3'-terminus. The introduction of the NcoI site, generating unique sticky ends, resulted in the addition of a GCG codon (encoding alanine) following the START codon. The PCR fragment obtained was digested with both NcoI and SalI REase and cloned into a pRZ4737 vector digested with compatible BspHI and SalI REases [35] to form a pRZ-TspDTI clone.

### Expression of the *tspDTIRM *gene under P_R _promoter in *E.coli *and purification of the recombinant TspDTI RM enzyme

The pRZ-TspDTI clones were subjected to protein expression experiments. *E. coli *DH11S [pRZ-TspDTI] was subjected to mini- scale expression in 50 ml TB media supplemented with chloramphenicol and maltose at 28°C with vigorous aeration, followed by PR promoter induction by a temperature shift to 42°C when OD_600_ reached 0.7. The culture growth was continued for 12 h at 42°C. Uninduced control and induced cells were subjected to SDS/PAGE, and gels were analysed for the appearance of the expected band size of approx. 126 kDa and for endonucleolytic activity in crude lysates.

Expression of *tspDTIRM *in *E. coli *DH11S [pRZ-TspDTI] was initiated with bacteria inoculum washed out from a Petri dish into 1 L of rich TB media, supplemented with chloramphenicol at 28°C. The culture was grown with vigorous aeration until OD_600_ reached 0.3; then the culture was transferred to a fermentor vessel containing 9 L of the media and grown until OD600 was 0.6. Induction was achieved with a rapid temperature increase to 42°C by the addition of 7 L of the medium warmed to 70°C, and growth was continued for 17 h at 42°C. Once an OD600 of 2.0 was reached, the culture was cooled down to 4°C and the cells were recovered by centrifugation. The purification steps used were as we have described previously for the native enzyme [20], with the following modifications:

1. *Polyethyleneimine (PEI) *removal of nucleic acids was performed with a bacterial pellet suspended in buffer A1 (50 mM Tris-HCl pH 7.0; 150 mM NaCl; 5 mM EDTA; 5 mM βME; 0.1% Triton-X-100, 1 mM AEBSF and 20 μg/ml benzamidine).

2. *Phosphocellulose chromatography *was conducted in buffer B (20 mM K/PO_4_ pH 7.2 at 25°C, 100 mM NaCl, 0.5 mM EDTA, 0.02% Triton X-100, 0.02% Igepal, 0.02% Tween, 5 mM βME, 1 mM AEBSF, 20 mg/ml benzamidine).

3. *Hydroxyapatite chromatography was used as the third chromatographic step in *buffer C (20 mM K/PO_4_ pH 7.2 at 25°C, 100 mM NaCl, 0.02% Triton X-100, 0.02% Igepal, 0.02% Tween, 5 mM βME, 1 mM AEBSF, 20 mg/ml benzamidine).

4. *DEAE-Sephadex chromatography *was used as the fourth step, using buffer D (20 mM Tris-HCl pH 7.5 at 25°C, 70 mM NaCl, 0.02% Triton X-100, 0.02% Igepal, 0.02% Tween, 5 mM βME, 1 mM AEBSF, 20 mg/ml benzamidine).

5. Heparin-agarose chromatography was used as the fifth step, using buffer D.

6. *Molecular sieving *was omitted.

### REase and MTase assays

For REase assays various conditions were used, depending on the experiment. The reactions were performed in 50 μl of 'primary TspDTI REase' buffer (10 mM Tris-HCl pH 8.5 at 25°C; 1 mM DTT), supplemented with appropriate additives and DNA substrates.

One unit of the TspDTI REase is defined as the amount of enzyme required to hydrolyse 1 μg of pUC19 in 1 h at 70°C in 50 μl of 'primary TspDTI REase' buffer, enriched with 10 mM MgCl_2_ and 50 μM SAM, resulting in a stable partial cleavage pattern.

The *in vitro *modification activity of TspDTI enzyme was tested by the DNA protection assay, where 0.5 μg of 390 bp PCR DNA fragment was used as a substrate in 50 μl of TspDTI MTase buffer (10 mM Tris-HCl pH 8.5; 1 mM DTT; 200 μM SAM) supplemented either with10 mM CaCl_2 _or with 10 mM EDTA. After addition of TspDTI protein, the reaction mixture was incubated for 16 h at 70°C. Proteinase K was added to the solution and the incubation continued for an additional 60 min at 55°C. Samples were purified to remove all traces of proteins and divalent cations from the methylation reaction mixture, and the resulting DNA was challenged with an excess of TspDTI (2 : 1 molar ratio of enzyme to recognition sites) for 1 h in 50 μl of 'primary TspDTI REase' buffer supplemented with 10 mM MgCl_2 _at 70°C. The reaction products were then resolved by agarose gel electrophoresis.

### Bioinformatics analyses

Sequence searches of the REBASE database of RM systems were done with the BLAST utility at the REBASE website [2]. Further searches of the GenBank database were carried out at NCBI with PSI-BLAST [38] with a conservative e-value threshold of 1E-30. Multiple sequence alignment of TspDTI homologues retrieved from GenBank was calculated with MUSCLE [39]. Identification of matches between TspDTI and proteins with known structures was carried out using the GeneSilico MetaServer [30], which is a gateway for a variety of bioinformatics methods, in particular those for the prediction of secondary structure and structurally disordered regions, and also for protein fold-recognition (FR) analysis. Previously, this method was successfully used to predict the structures of several nucleases, for which subsequent crystallographic analyses confirmed the validity of predictions [40-42]. Alignments between the TspDTI sequence (or its fragments) and sequences of proteins of known structure reported by the FR methods were compared, evaluated and ranked by the PCONS method [43].

## Competing interests

Arvydas Lubys, Danute Ramanauskaite, Goda Mitkaite are affiliated with Thermo Fisher Scientific Inc. (USA), Fermentas branch (Vilnius, Lithuania) and provided scientific information concerning TsoI amino acid sequence and selected enzyme features. The authors declare that they have no competing interest.

N-terminal TspDTI proteolytic fragments sequencing and *tspDTIRM *gene sequencing were supported by EURx Ltd. (Gdansk, Poland), and the data obtained were deposited in the GenBank (EF095489.1; 31-OCT-2006; Skowron, P.M. and Zylicz-Stachula, A.). The DNA and proteolytic fragments sequencing data were re- confirmed in the course of this project. The authors declare that they have no competing interest.

## Authors' contributions

AZS performed most of the experiments, participated in the design and interpretation of all the experimental analyses, prepared most of the figures and drafted the manuscript. OZ performed some experiments. JMB carried out the sequence analysis and bioinformatics studies, prepared Figures 4 and 5, wrote the bioinformatics section and helped to draft the manuscript. AL conceived and coordinated the TsoI project. DR and GM performed the TsoI-related experiments. PMS conceived the project, came up with the concept of the new *Thermus *sp. enzyme family, participated in the design and interpretation of the experiments and drafted the manuscript. All the authors read and approved the final manuscript.

## Supplementary Material

Additional file 1 **Scheme of sequencing and cloning of the *tspDTRM* gene**. Click here for file

Additional file 2 **DNA and amino acid sequence of the *tspDTIRM* gene and its flanking regions**. The predicted amino acid sequence of the 126.9 kDa TspDTI protein is indicated in capital letters. The DNA sequences of the flanking regions are indicated in italics. The internal amino acid sequences of the TspDTI enzyme, determined by chemical analysis of proteolytic fragments, are underlined. The ATG start codon is in bold. The TGA stop codon is shown in red. The potential TspDTI Ribosome Binding Sites (RBS) are boxed and in italics. The crucial amino acids of the catalytic centres are dark red, bold and underlined. Click here for file

Additional file 3 **DNA and amino acid sequence of the *tsoIRM* gene and its flanking regions**. The predicted amino acid sequence of the 1116 amino acids/126.5 kDa TsoI protein as based on cloned tsoIRM gene is shown in capital letters (manuscript in preparation). The crucial amino acids of the catalytic centres are dark red, bold and underlined. Click here for file
