# Supplementary figures and images for: Related bifunctional restriction endonuclease-methyltransferase triplets: TspDTI, Tth111II/TthHB27I and TsoI with distinct specificities
Source: BMC Mol Biol. 2012 Apr 10;13:13. doi: 10.1186/1471-2199-13-13 (PMC3384240; doi:10.1186/1471-2199-13-13)

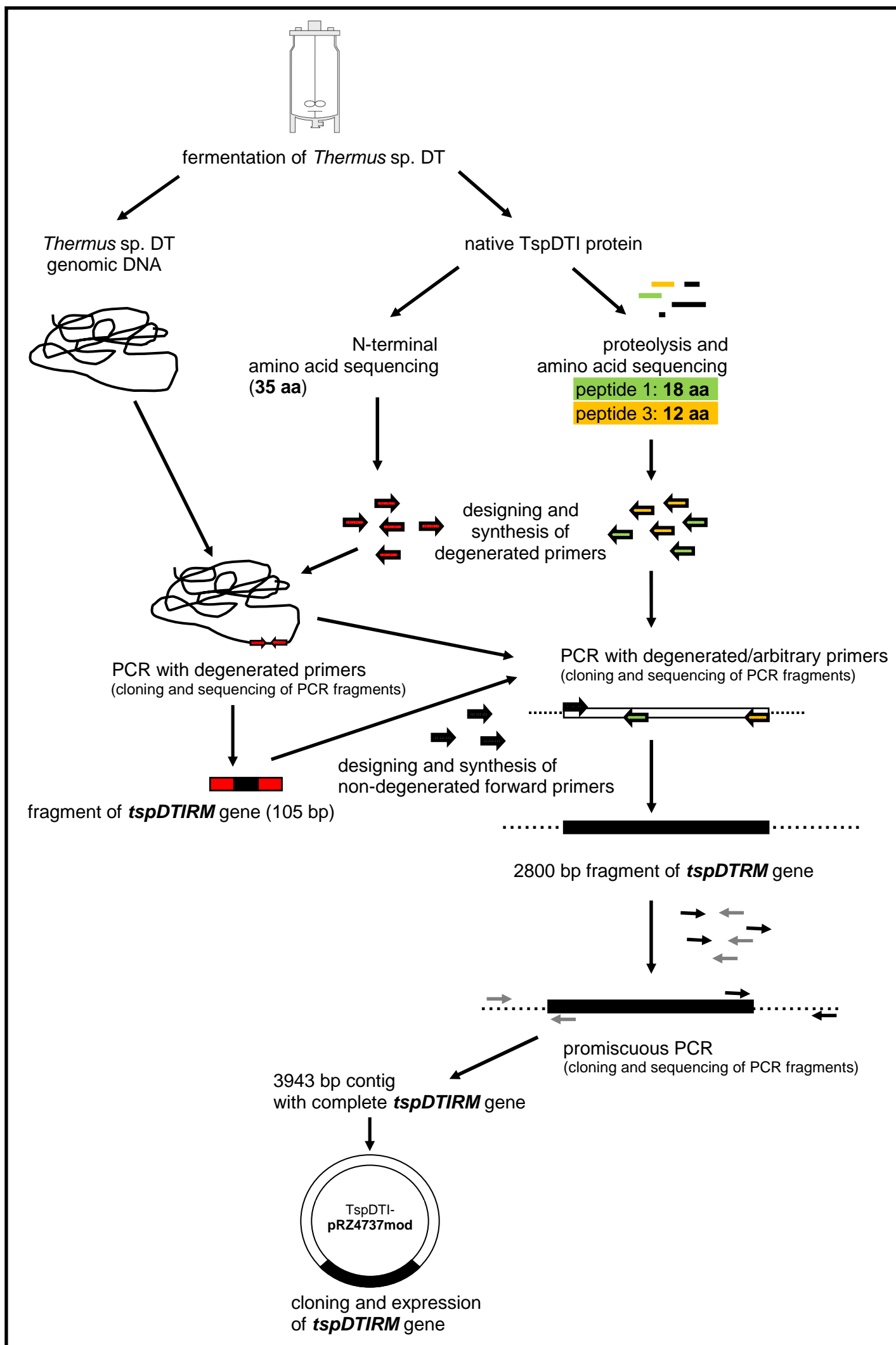

Supplement: Additional file 1 — Scheme of sequencing and cloning of the tspDTRM gene. [file 1471-2199-13-13-S1.PDF]
