# Supplementary material for: Related bifunctional restriction endonuclease-methyltransferase triplets: TspDTI, Tth111II/TthHB27I and TsoI with distinct specificities
Source: BMC Mol Biol. 2012 Apr 10;13:13. doi: 10.1186/1471-2199-13-13 (PMC3384240; doi:10.1186/1471-2199-13-13)
Supplement: Additional file 2 — DNA and amino acid sequence of the tspDTIRM gene and its flanking regions. The predicted amino acid sequence of the 126.9 kDa TspDTI protein is indicated in capital letters. The DNA sequences of the flanking regions are indicated in italics. The internal amino acid sequences of the TspDTI enzyme, determined by chemical analysis of proteolytic fragments, are underlined. The ATG start codon is in bold. The TGA stop codon is shown in red. The potential TspDTI Ribosome Binding Sites (RBS) are boxed and in italics. The crucial amino acids of the catalytic centres are dark red, bold and underlined. [file 1471-2199-13-13-S2.PDF]

*TTT GTG GTC ACC CTC ACT TCA AGA TTT TAG AAT AGG GAT GTT ATA ATA GGA GAA AAT GTG* -151

*CCT ATT AAG GAG AAC CTT CCA AAC CCT TTT AAA ACA TCC TCA TGA GGC TTT TGT TGT ATA* -91

*GCC CTC CTT AGA AGG GTG TAT ATT AAA CGT GTC TTT CAA GGC GCT GTG CTT GCC CGT GTG* -31

**RBS**

*GCC AAG GTC GGG ATT AGA AAT AGT GGC AAT* **ATG** AGC CCT TCC AGG GAA GAA GTT GTT GCC 30

**M** S P S R E E V V A 10

CAT TAC GCT GAC AGG CTT CAC CAA GTT CTT CAG AAA ACC ATA GCG CAG AAC CCC AAC GAG **90**

H Y A D R L H Q V L Q K T I A Q N P N E **30**

GCT GAG TTC CGA AGA GCG GTA GAG CCT CTA TTG GAG GAG TTT TTA CGG GAA ATG GGC CTC **150**

A E F R R A V E P L L E E F L R E M G L **50**

GAG CCC CTA GCC CGA GCA GAG TAC ACC TTA GCA CAG GGA CGG GCC GAT GCC ATC TTT AAT **210**

E P L A R A E Y T L A Q G R A **D** A I F N **70**

**PD-(D/E)XK: catalytic center of REase**

CGC TTG GTC ATC GAG TAC GAG CGC CCC GGT GTC CTC AAG CCT AGG CCG GAC GCT GCC ACA **270**

R L V I **E** **Y** **E** R P G V L K P R P D A A T **90**

CGG CAT GCG GTG CAA CAG GTT AAG GAT TAC CTG TCC GGC ATC GCC CAG AGG GAG CGG CAT **330**

R H A V Q Q V K D Y L S G I A Q R E R H **110**

GCC AAG GAA CGC CTA GCA GGT GTG GCG TTT GAC GGG CGC TAT TTG ATC TTC GTC CGG CAC **390**

A K E R L A G V A F D G R Y L I F V R H **130**

ATG GGG GAA AGG TGG GTG GAG GAA CCT CCC GTG GAG GCC AAT CCC CAC TCG CTC AAG CGC **450**

M G E R W V E E P P V E A N P H S L K R **150**

TTC CTC ACC TGG CTT GCG GGC TTG GCC TCG GGG ATT GCC CTG ACC TCG GAA AAC CTT AAC **510**

F L T W L A G L A S G I A L T S E N L N **170**

CGG GAC TTC AGC ATA GAG CAA TTG CGC ACC CAA ACC ATC CTC CGA GGG CTT TAC CAA GCC **570**

R D F S I E Q L R T Q T I L R G L Y Q A **190**

TTG GAA AAG GCC TTG GCG AGA AAG GGT CTG GTC CGC CAG CTC TTT GAG CAA TGG CGC ATC **630**

L E K A L A R K G L V R Q L F E Q W R I **210**

TTC TTC AGC GAG GCC ATA GAC TAC TCC GAA ACC TTT GGC GGG CGC AAG CTG GAA CCC CTT **690**

F F S E A I D Y S E T F G G R K L E P L **230**

AAA AAG TGG GTG CGC AAA GCG GGC CTG GAC ATC CAA ACC CCT GAA GAG GCC GAG CGC TTC **750**

K K W V R K A G L D I Q T P E E A E R F **250**

TTT TTC GTT CTG CAC ACG TAT TTT GCC CTG CTG GCC AAA CTT CTT GCC TGG CTG GCC CTT **810**

F F V L H T Y F A L L A K L L A W L A L **270**

TCC CGC CAC ATG GGG GTT AGG CTG GGA GCC CCA GTG TTC TCT GCC CTT GCT GCC GCA GAC **870**

S R H M G V R L G A P V F S A L A A A D **290**

GGG GAA ACC CTG CAG AAG AGG CTT GGG GAG ATG GAG TCG GGA GGC ATA TTC CGG CAG TAC **930**

G E T L Q K R L G E M E S G G I F R Q Y **310**

GGC ATC CTC AAC CTT CTG GAG GGG GAT TTC TTC GCC TGG TAC CTT CAC GCC TGG AGC AGC **990**

G I L N L L E G D F F A W Y L H A W S S **330**

GAG GTG GAG CGC GCC CTC CGT GCC CTC ATC GAA CGC CTA GAT GAG TAC GAC CCC ACC ACC **1150**

E V E R A L R A L I E R L D E Y D P T T **350**

CTC TCC CTC TTC CCC GAG GAA ACC CGG GAC CTT TTC AAA AAG CTC TAT CAC TAC CTC TTA **1110**

L S L F P E E T R D L F K K L Y H Y L L **370**

CCC CGG GAA ATA CGC CAC AAC CTG GGG GAG TAC TAC ACA CCG GAT TGG CTC GCA TGG CGC **1170**

P R E I R H N L G E Y Y T P D W L A W R **390**

CTC CTG GTG CAG CTG GAC AAT ACC TTT TTC GCC GGA ACC CCC TCG CCC AAT GAC GAA AAG **1230**

L L V Q L D N T F F A G T P S P N D E K **410**

**Motif I: S-adenosylmethionine-binding site**

TTA CGC CAG AAG CTC CTT AGC ACC CGA TTT TTA GAC CCC GCA TGC GGA TCC GGT ACT TTC **1290**

L R Q K L L S T R F L **D** **P** **A** **C** **G** **S** **G** T F **430**

CCG GTC CTT GTC ATC GGA AGG ATG CTG GAG TTG GGC AGA TTG TTG ATG GTT CCG GAG AGG **1350**

P V L V I G R M L E L G R L L M V P E R **450**

GAC CTC TTA GAA GCC ATC CTC AAG AAC GTG GTG GGC TTC GAC CTC AAC CCC CTG GCC GTC **1410**

D L L E A I L K N V V G F D L N P L A V **470**

CTC ACG GCA CGG GTG AAC TAT CTA CTG GCC ATT TCC GAC CTC CTT CAG TAC CGC CAG GGG **1470**

L T A R V N Y L L A I S D L L Q Y R Q G **490**

GAT ATC ACC ATA CCC ATT TAC CTG GCC GAC TCG GTC CGT ACA CCC GCC GAG GGG CAG GAC 1530
D I T I P I Y L A D S V R T P A E G Q D 510

CTT TTT AGC CAG GGC ATC TTC GTC TTC CCT ACC GCG GTG GGT GAT TTC CAG GTG CCG GCA 1590
L F S Q G I F V F P T A V G D F Q V P A 530

GCT CTG GTG ACT GCC CCT AAA CGT TTC GAC CGC TTC TGC GAG ATC TTG GAA AGC AGC ATC 1650
A L V T A P K R F D R F C E I L E S S I 550

CGG TCC GAG GTA GAC CCG CAA GCC TTT TTG GAA CGC ACC CGC AGG GAG CTG GAC CTT AAC 1710
R S E V D P Q A F L E R T R R E L D L N 570

CCA AGC GAA TGG GAT GAC AAT GCC CGT AAG CTG GCT GAG GAG CTT TAT ACC AAG CTT CTA 1770
P S E W D D N A R K L A E E L Y T K L L 590

GAC CTT CAC AGA AGA GGT CTC AAC GGC CTA TGG GCG CGG CTT CTC AAG AAC AAC TTC GCC 1830
D L H R R G L N G L W A R L L K N N F A 610

**Motif IV: catalytic center of MTase**

CCC CTA ACT GTG GGA CGG TTT GAC TAC ATC GTG GGC AAC CCG CCC TGG ATT AAC TGG GAA 1890
P L T V G R F D Y I V G N P P W I N W E 630

CAC CTG CCC GAT GAA TAC CGT GAA AGC ATC AAA CAC CTC TGG CTC CGT TAC CGG ATA GCT 1950
H L P D E Y R E S I K H L W L R Y R I A 650

GGC TCC TAC ACA GGT GGG CGT CCC CGG CTC GGA GCG GTC AAG GTG GAC ATA TCC GCC CTC 2010
G S Y T G G R P R L G A V K V D I S A L 670

ATG ACC TAC ATC GTG GTT GAC AAC TTG CTC AAA AAT GGC GGC AAA CTG GGC TTC GTC ATC 2070
M T Y I V V D N L L K N G G K L G F V I 690

ACC CAA TCG CTC CTG AAA ACT GCA GCG GGG GCT GGA TTC CGG CGC TTA AGC ATC CCT ACG 2130
T Q S L L K T A A G A G F R R L S I P T 710

GAA AAG GGC AAG GAA GTC CCT CTT CGC ATT GTG TAC GTG GAC GAC ATG GTG GAC CTC AAC 2190
E K G K E V P L R I V Y V D D M V D L N 730

CCC TTT GAA GGT GCT TCT AAC CGA ACC GCT GTA ATC GTG CTA GAG AAA GGT AAG CCA ACC 2250
P F E G A S N R T A V I V L E K G K P T 750

CAG TAC CCC GTG CCC TAT ACC GTG TGG CGC AAG AAC AGG GGC GTG CGC TTC ACC TAC GAT 2310
Q Y P V P Y T V W R K N R G V R F T Y D 770

AGC ACC CTG GAG GAC GTA CAG GCT GCC ACA CGC CGG CTC CAG TTC CAG GCC CAA CCC GTG 2370
S T L E D V Q A A T R R L Q F Q A Q P V 790

GAC CCC GAC AAC CCC ACC TCT CCC TGG CTC ACC GCT CGC CCG AAG GCG CTG CGC GCC GTG 2430
D P D N P T S P W L T A R P K A L R A V 810

CGC AAA ATC CTA GGC CAG TCC GAC TAT GAG GCT CAC GCT GGC GTA TAC ACC GGA GGG GCA 2490
R K I L G Q S D Y E A H A G V Y T G G A 830

AAT GCC GTT TAC TGG GTA GAA CCA GTA CTG AAA CGC CCC GAT GGG CTC TGG GTA GTG CGC 2550
N A V Y W V E P V L K R P D G L W V V R 850

AAC CTG ACC GAA GGG GCC AAG GTG AAG GTA GAG GAG GTA ACC GAA ACC ATT GAG CCT GAT 2610
N L T E G A K V K V E E V T E T I E P D 870

CTT CTC TAT CCA CTC CTT CGT GGC CGC GAT GTG CAG CGC TGG CGG GCT GAA CCT TCA GCG 2670
L L Y P L L R G R D V Q R W R A E P S A 890

TGG ATA ATC ATC CCG CAG GAC CCT AAC AAC CCA AGC CGG GCC TAT CCG GAG GCA AAA CTC 2730
W I I I P Q D P N N P S R A Y P E A K L 910

AAA GTT GAC TAT CCT AGA CTT TAT GCC TAC CTT AAG CAA TTT GAA TCC GTA CTA CGC CGA 2790
K V D Y P R L Y A Y L K Q F E S V L R R 930

CGG GCT GCT TAT CAG CAG ATC CTT TGC AAG CGT GAG CCT GAG TTT TAC GGA ATC ATG GAC 2850
R A A Y Q Q I L C K R E P E F Y G I M D 950

ATT GGC CAC TAC TCC TTC TCC CCC TGG AAG GTG GTG TGG ACA AGG CTC GCC AAG ATT GAG 2910
I G H Y S F S P W K V V W T R L A K I E 970

GCG GCG GTG GTT GGC TTG CAT GGG AGA AAA CCA GTC ATA CCA CAA GAA ACC GTC AGC TTG 2970
A A V V G L H G R K P V I P Q E T V S L 990

GTT CAA TGT GAC ACG AAA GAA GAG GCA TAC TAT ATC GCC GCG TTA GTC AAC TCG ACG GCC 3030
V Q C D T K E E A Y Y I A A L V N S T A 1010

TTC CAG TTA GCC GCT ACC TCC TAT AGC CAA GAG GGT GGC AAA AGC ATG GGA TCT ATG CAC 3090
F Q L A A T S Y S Q E G G K S M G S M H 1030

| | | | | | | | | | | | | | | | | | | | | |
|---|---|---|---|---|---|---|---|---|---|---|---|---|---|---|---|---|---|---|---|---|
| ATC | CTC | GAG | CAC | ATC | CGC | ATT | CCA | CGC | TAC | CAA | CCC | ACC | GAC | CCC | GTA | CAT | CGG | CGG | CTT | **3150** |
| I | L | E | H | I | R | I | P | R | Y | Q | P | T | D | P | V | H | R | R | L | **1050** |
| GCG | GAG | CTG | TCG | CAG | GCG | GCG | CAC | GAA | GCG | GCC | CAG | GCG | GGA | GAT | GAA | AAG | CGC | TTA | GAG | **3210** |
| A | E | L | S | Q | A | A | H | E | A | A | Q | A | G | D | E | K | R | L | E | **1070** |
| GCC | CTG | GAG | ACA | GAA | ATC | GAC | CGG | GAG | GCG | GCT | AAG | CTT | TGG | GGC | CTG | ACG | GAA | GCC | GAG | **3270** |
| A | L | E | T | E | I | D | R | E | A | A | K | L | W | G | L | T | E | A | E | **1090** |
| CTC | AGA | GAG | ATT | CAA | GAG | AGC | CTA | CGG | GAG | CTG | GAG | GGA | GAG | GTA | CCT | GCA | GCA | GAG | GAA | **3330** |
| L | R | E | I | Q | E | S | L | R | E | L | E | G | E | V | P | A | A | E | E | **1110** |
| GAA | GCA | **TGA** | *GGG* | *CTC* | *TTC* | *AAG* | *ATT* | *TGC* | *GGC* | *ACG* | *TCC* | *TGA* | *AAA* | *CCC* | *TTG* | *CCC* | *AGG* | *AAG* | *CAG* | **3390** |
| E | A | ***** | | | | | | | | | | | | | | | | | | **1112** |
| *AGG* | *TGC* | *TCT* | *CTG* | *CTG* | *TGG* | *GTG* | *GCG* | *CGG* | *TGG* | *AGG* | *ACC* | *TCG* | *AGC* | *GGC* | *CTG* | *ATT* | *TTG* | *AGG* | *AAC* | *3450* |
| *GGG* | *AGG* | *ACC* | *AGG* | *CCC* | *TCA* | *ACG* | *AAG* | *AGG* | *AGC* | *GGG* | *TTT* | *TCG* | *AAA* | *TTA* | *TCT* | *TTG* | *AGA* | *AAA* | *GCT* | *3510* |
| *TGG* | *ATG* | *GAA* | *CCA* | *AAC* | *CAA* | *GCC* | *TCT* | *ACC* | *GCC* | *CCA* | *CCC* | *CAC* | *CTT* | *TAA* | *GCC* | *GCA* | *GCA* | *AGA* | *GGC* | *3570* |
| *ATC* | *TTT* | *TTA* | *GCT* | *ATT* | *TCC* | *TGG* | *ATG* | *GCT* | *CCT* | *TCC* | *GCT* | *CCT* | *ACT* | *TCC* | *TGG* | *GTA* | *CCC* | *TCC* | *TGG* | *3630* |
| *AAC* | *ACG* | *AGC* | *GGG* | *AAA* | *CCC* | *CCG* | *TGC* | *ACT* | *TTG* | *CCC* | *AAA* | *TAG* | *GGG* | *CAT* | *GCG* | *TGC* | *TTC* | *GCC* | *GCG* | *3690* |
| *AGG* | *ATA* | *ATG* | *GCT* | *CGG* | *TGC* | *GGC* | *GTA* | *GTA* | *CGG* | *ATT* | *CAA* | *ATT* | *GCT* | | | | | | | *3732* |
