# Supplementary material for: Related bifunctional restriction endonuclease-methyltransferase triplets: TspDTI, Tth111II/TthHB27I and TsoI with distinct specificities
Source: BMC Mol Biol. 2012 Apr 10;13:13. doi: 10.1186/1471-2199-13-13 (PMC3384240; doi:10.1186/1471-2199-13-13)
Supplement: Additional file 3 — DNA and amino acid sequence of the tsoIRM gene and its flanking regions. The predicted amino acid sequence of the 1116 amino acids/126.5 kDa TsoI protein as based on cloned tsoIRM gene is shown in capital letters (manuscript in preparation). The crucial amino acids of the catalytic centres are dark red, bold and underlined. [file 1471-2199-13-13-S3.PDF]

M S P S R E E V V A H Y A D R L H Q V L 20

Q K T I A Q N P N E A E F R R A V E P L 40

L E E F L R E M G L E P L A R A E Y T L 60

PD-(D/E)XK: catalytic center of REase

A Q G R A **D** A I F N R L V I **E** **Y** **E** R P G 80

V L K P K P D A A T R H A V Q Q V K D Y 100

L S G I A Q R E R H A K E R L A G V A F 120

D G R Y L I F V R H M G E R W V E E P P 140

V E A N P H S L K R F L T W L A G L A S 160

G I A L T S E N L N R D F S I E Q L R T 180

Q T I L R G L Y Q A L E K A L A E E G L 200

V R Q L F E Q W R I F F S E A I D Y S E 220

T F G G R K L E P L K K W V R K A G L H 240

I Q T P E E A E R F F F V L H T Y F A L 260

L A K L L A W L A L S R H M G V R L G A 280

P V F S A L A A A D G E T L Q K R L G E 300

M E S G G I F R Q Y G I L N L L E G D F 320

F A W Y L H A W S S E V E R A L R A L I 340

E R L D E Y D P T T L S L F P E E T R D 360

L F K K L Y H Y L L P R E I R H N L G E 380

Y Y T P D W L A W R L L V Q L D N T F F 400

A G T P S P N D E K L R Q K L L S T R F 420

Motif I: S-adenosylmethionine-binding site

L **D** **P** **A** **C** **G** **S** **G** T F P V L V I G R M L E 440

L G R L L M V P E R D L L E A I L K N V 460

V G F D L N P L A V L T A R V N Y L L A 480

I S D L L Q Y R Q G D I T I P I Y L A D 500

S V R T P A E G Q D L F S Q G I F V F P 520

T A V G D F Q V P A V L V T A P K R F D 540

R F C E I L E S S I R S E V D P Q A F L 560

E R T R R E L D L N P S E W D D N A R K 580

L A E E L Y T K L L D L H R R G L N G L 600

W A R L L K N N F A P L T V G Q F D Y I 620

Motif IV: catalytic center of MTase

V G **N** **P** **P** **W** V N W E H L P D N Y R R S I 640

A P L W A R Y E I F S H K G F D A I L G 660

K S K D D I S V L M T Y T V M D K L L K 680

D K G R L G F V I T Q S V F K T G G G G 700

Q G F R R F R I P Q G K E Q F T P L A V 720

I H V D D M V D L N P F E G A S N R T A 740

V M V L E K G K P T K Y P V P Y T V W R 760

K K R G T R F T Y D S T L D E V L S A T 780

R R L H F F A E P V D P K D P T S P W L 800

T A R P K V L K A V R K I L G K S D Y R 820

A R K G V T P S V N S V F W L I E A L K 840

R P D G L I L I H N L T E G A K V E V G 860

E V S E T I E P D L L Y P L L R G R D V 880

K R W R A E P S A M I L I T H E P G M R 900

L K A I P E K E M Q T R Y P R T Y G Y L 920

K R F E Q V L R R S A V F R R Y F I R K 940

K G G E L V D A G P F Y S M F N V G D Y 960

T F A P W K V V W R E I A S D L T A A V 980

V S S R E G K T I V P D H K L V L V A C 1000

S S D I E A H F I C A L L N S S I V R F 1020

V A L G Y A I Q T Q F A P H L L D F I R 1040

I P R Y N P T D P L H R R L S E L S Q A 1060

A H K A A Q A G D E K R L E A L E A E I 1080

D R E A A K L W G L T E A E L R E I Q E 1100

S L R E L E G E V P A A E E E A * 1116
